# A protocol for a new methodological model for work-related shoulder complex injuries: From diagnosis to rehabilitation

**DOI:** 10.1186/s12891-017-1435-2

**Published:** 2017-02-07

**Authors:** Igor Setuain, Miriam Gonzalez-Izal, Ainara Paularena, Jose Luis Luque, Lars L. Andersen, Mikel Izquierdo

**Affiliations:** 1 Department of Health Sciences, Public University of Navarra, Pamplona, Navarra Spain; 2 Mutua Navarra, Medical Assistance Inssurance Society, Pamplona, Navarra Spain; 3 National Research Centre for the Working Environment, Copenhagen, Denmark; 4 Department of Health Science and Technology, Physical Activity and Human Performance group, SMI, Aalborg University, Aalborg, Denmark

**Keywords:** Shoulder, Work environment, Injury, Management

## Abstract

**Background:**

Work-related injuries of the shoulder complex represent a challenge for clinicians because of the large variety of clinical entities involved and the broad anatomic structures that can be affected. Furthermore, commonly performed orthopedic tests have demonstrated limited accuracy for diagnosing the injury despite considerable research efforts. The aim of this study protocol is therefore to describe a comprehensive approach integrating both a clinical- and functional status-based pathology and an adapted rehabilitation prescription.

**Methods/Design:**

A longitudinal cohort study will be performed at the Department of Rehabilitation and Medical Assistance of a mutual insurance society for work-related injury management in Spain (Mutua Navarra, Pamplona, Navarra Spain). Patients will be attended by an occupational physician who specializes in work-related injuries and is part of the project team that will systematically visit all the participants. After the medical diagnosis and any requested supplementary evaluations (i.e., radiological examinations), the patients will be referred to the rehabilitation service. Before the physiotherapeutic rehabilitation program is initiated, the patients will undergo a comprehensive functional screening at the biomechanics laboratory. Using a decision-making scheme, the identified functional deficits will be used to customize the individual rehabilitation plan.

**Discussion:**

The proposed objective criteria-based shoulder diagnosis and rehabilitation model could be a new effective strategy for minimizing the time required to regain functional capacity and recover from symptoms among patients with work-related shoulder injuries.

**Trial registration:**

The study protocol has been registered on Clinical Trials.gov as NCT02732002 (April 10^th^ 2016).

**Electronic supplementary material:**

The online version of this article (doi:10.1186/s12891-017-1435-2) contains supplementary material, which is available to authorized users.

## Background

Persistent shoulder pain and functional impairments are common among patients with shoulder injuries, accounting for 5% of all medical practice visits [1]. As reported by Ginn et al. 2005 [2], the shoulder is the fourth most common site of musculoskeletal pain reported by patients visiting physicians or physical therapists, exceeded only by the neck, knee and back regions. Causal relationships were recently reported by B Larsson et al. 2007 [3] between forceful exertions, a high level of static contractions, prolonged static loads, extreme postures, and combinations thereof and work-related disorders of the shoulder. Stressful working postures, e.g., working with the arms above shoulder height, have also been shown to increase the risk of long-term sickness absence in the general working population [4].

Shoulder pain can arise from different anatomical structures or a combination of several structures. Thus, subacromial or external impingement syndrome (SIS), rotator cuff pathology, superior labrum anterior posterior (SLAP) injury and glenohumeral (GH) ligament insufficiency are the most frequently cited anatomical pain-eliciting structures [5]. Other authors have described other clinical entities related to shoulder pathology, such as the so-called internal impingement syndrome and scapular dysfunction syndrome [6, 7]. Internal impingement syndrome is described as a compromise of the supraspinatus tendon against the greater tubercle of the humerus and the postero-superior glenoid rim [6]. Scapular dysfunction is defined as an alteration of scapular kinematics concomitant with rotator cuff impingement or disease and is characterized by a lack of posterior tilting, upward rotation and increased internal rotation or medial rotation of the scapula [7].

Several orthopedic tests are commonly performed to identify the clinical entity causing the pain. In short, these tests involve moving the affected arm into different positions, which are believed to irritate different shoulder structures [3, 6]. Depending on which testing position reproduces the greatest pain level, a clinical diagnosis is based on the link between the reproduced arm posture and the biological structure being stressed. For a more comprehensive explanation of the most frequently utilized orthopedic tests in the practical setting, the reader is referred to the following reports [3, 6, 8]. However, there is a lack of isolated orthopedic tests with sufficient sensitivity and specificity to diagnose and justify the commonly observed clusters of symptoms in many shoulder patients [6, 9]. Various authors have therefore focused their research on this issue, concluding that clustering orthopedic tests in relation to isolated clinical entities would improve their diagnostic accuracy [9, 10].

Several biomechanical models of shoulder function in the presence or absence of shoulder injury [11–16] have been developed. However, less is known regarding the standard of reference for the rehabilitation process. In this context, exercise therapy represents the most effective treatment for shoulder injury rehabilitation in terms of both symptomatic relief and function restoration [15–18]. In fact, numerous studies have assessed the effectiveness of exercise therapy versus other treatment modalities and have described the biomechanical (kinetic description) or neuromuscular (EMG activity) rationale for treating different causes of shoulder pain. For work-related shoulder pain, strength training in particular has demonstrated promising results for reducing pain and improving function [19, 20].

However, in the authors’ opinion, a comprehensive rehabilitation model for shoulder injuries that integrates the clinical entity and functional status and provides an individualized exercise rehabilitation program based on all the information obtained from both clinical and functional examinations is lacking. The purpose of the present study protocol is therefore to establish a comprehensive clinical model for shoulder injury management that integrates both clinical and biomechanical assessments and targets individual deficits in the posterior rehabilitation program. The present study protocol will be implemented by a non-lucrative public medical insurance corporation that functions as a satellite of the national public health system in Spain.

If our hypothesis is correct, an objective criteria-based rehabilitation algorithm (OCBRA) for rehabilitation could become a new effective strategy for improving functional capacity and symptom recovery among patients with shoulder injuries. This intervention is designed to improve the rate of complete return to previous activity levels, for which a concise clinical diagnosis and functional status picture seems imperative.

## Methods/Design

### Background

#### Study design and setting

This longitudinal cohort study with a historical clinical cohort serving as control group will be conducted at the Department of Rehabilitation and Medical Assistance of a mutual insurance society for work-related injury management in Spain (Mutua Navarra, Pamplona, Spain). The patients will be attended by a physician trained in the protocol implemented during this research. Following medical diagnosis and supplementary evaluations when requested (i.e., radiological examination), the patients will be referred to the rehabilitation service. Before initiating the physiotherapeutic rehabilitation program, the patients will undergo comprehensive functional screening at the biomechanics laboratory. Using a decision-making scheme, the identified functional deficits will be used to customize an individual rehabilitation plan. The rehabilitation plan will be conducted at the physical rehabilitation facilities of the institution by any of the 7 physical therapists working in the area (Additional file 1). Every professional attending the patients with shoulder complaints will be trained in and familiarized with the injury management model being implemented in the rehabilitation department. The training period will last for nearly 6 months, during which one of the authors (I.S) will explain and later supervise the successful application of the OCBRA at each component level. This training will take place during weekly meetings in which the therapist and the instructors will review every laboratory test, the rehabilitation prescription and the execution of the rehabilitative routines.

This study has been approved by the Clinical Research Ethics committee of the Public University of Navarra in accordance with the Declaration of Helsinki (the approval numbers for the clinical trials are Gov register: NCT02732002; PRS, protocols registration and results system). All the participants will be informed of the objectives and risks of the intervention. All the participants will sign an informed consent. The subjects’ names will be coded to preserve their anonymity.

**Fig. 1:**
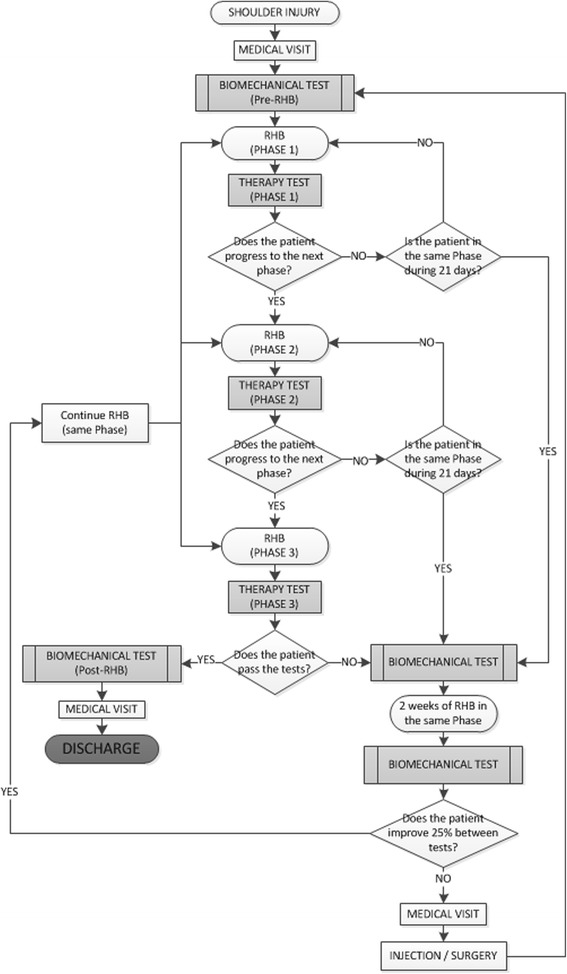
Multi-etiologic shoulder injury management model for rehabilitation algorithm description

The study decision-making scheme, i.e., the Objective Criteria Based Rehabilitation Algorithm (OCRBA), is presented in Table 1. For a clinically predominant pathology to be considered, at least 50% of the tests administered for each clinical entity must be positive. Every patient will be allocated to an injury type- and functional status-based rehabilitation program. During the rehabilitation process, the physical therapist will periodically evaluate the patient’s clinical status for stage progression during the procedure (Fig. 1). The criteria for periodical evaluation are based on the therapist’s subjective assessment of the patient’s progress over a 15-day period. Patients who do not progress to the next phase within 21 days will be referred to the laboratory for a biomechanical test. After 15 more days of the rehabilitation program in the same phase, another biomechanical test will be performed to evaluate whether the patient is making progress. If the patient does not improve by 25% between tests, the rehabilitation program will be considered ineffective, and the patient will be referred to the physician, who will propose other therapeutic modalities, such as joint corticoid infiltration or surgery (Fig. 1). This criterion is based on the empirical experience of the medical staff participating in the research and aims to avoid endless rehabilitation processes that reach a point of no return in the assistance chain and create economic burden for health care institutions. When the patient is clinically recovered, he or she will be referred by the physical therapist to the laboratory for a post-rehabilitation functional screening examination. Additionally, 50 patients recruited from the historical cohorts, will be recruited from the year before the OCBRA model is implemented to serve as controls. Concurrent randomization of patients is not possible in the present clinical research because every health institution has an ethical commitment to make the best effort to help patients recover from their illness.

**Table 1:** Exercise prescription according clinical predominant pathology and patient’s functional status. Every test is performed recording isometric dynamometry (except for the GIRD). The rehabilitation staff prescribes an individualized exercises according to the functional status of the patient (column 3), determined by the functional tests performed in the biomechanical laboratory, and according to the clinical predominant pathology (column 2), determined by the medical staff and the functional tests

CLINICAL PREDOMINANT PATHOLOGY	ORTHOPEDIC TEST PERFORMED	PATHOLOGY RELATED EXERCISES (mandatory)	FUNCTIONAL STATUS EXERCISES (optional depending on patient’s functional status)
Rotator Cuff Pathology	Jobe test Patte test Gerber test	Glenohumeral instability exercises Scapulothoracic instability exercises	Strength deficit exercises Internal rotation deficit exercises
Scapular Dyskinesis	Scapular Retraction test Lateral Slide Scapular test	Scapulothoracic instability exercises	Glenohumeral instability exercises Strength deficit exercises Internal rotation deficit exercises
Impingement (micro-instability)	Howkins test O’Brien test Internal impingement test	Scapulothoracic instability exercises Strength deficit exercises	Strength deficit exercises Internal rotation deficit exercises
Instability	Apprehension test	Glenohumeral instability exercises	Scapulothoracic instability exercises Strength deficit exercises Internal rotation deficit exercises
Biceps-SLAP pathology	Speed test Upper cut test Biceps load test	Glenohumeral instability exercises Strength deficit exercises	Scapulothoracic instability exercises Internal rotation deficit exercises
GIRD	Sleeper test	Internal rotation deficit exercises	Glenohumeral instability exercises Scapulothoracic instability exercises Strength deficit exercises

Full recovery will be determined by negative results on the last medical examination (for both the patients in the OCBR group and the clinical cohorts serving as controls) and positive results on the biomechanical evaluation (only for the patients in the OCBR algorithm). As the clinical cohorts will not undergo functional evaluations, the time to full recovery and the number of physician and physiotherapy visits will be analyzed to compared the cost-effectiveness of the two injury management models (the proposed vs. previous typical care).

#### Study participants and eligibility criteria

Every Spanish-speaking patient over 18 years of age seeking treatment from a physician at our institution due to work-related shoulder complaints of a mechanical origin (i.e., related to movement repetitions at work) lasting more than 6 weeks will be eligible for our rehabilitation model. This preliminary time-based criterion for exclusion from the program is designed to prevent the inclusion of transient shoulder ailments that could adequately resolve with rest and NSAIDs (non-steroidal anti-inflammatory drugs).

### Detailed description

#### Shoulder multifactorial management intervention group

##### Patient status-based rehabilitation model construction

The clinical reasoning for patient investigation using the rehabilitation model is adapted from previously published data by Cools et al. [6]. These authors made a brilliant effort to generate a clinical reasoning algorithm to guide the clinician during the process of diagnosing shoulder impingement-related injuries. In the cited study, the authors clustered different orthopedic tests to differentiate between shoulder impingement, instability, SLAP and/or posterior capsule tightness. Thus, several tests were performed to diagnose or rule out the same clinical entity in an attempt to improve the diagnostic accuracy of the physical examination [6, 12].

Thus, in the present study, we aim to complement the model in two different ways. First, we will record the quantitative variables of ROM (°) and force output (N) for each of the orthopedic tests performed (see the primary outcome measures section for more details). This protocol supplementation will reinforce the posterior rehabilitation program by addressing the specific muscle force deficits observed during the complete evaluation, thus complementing the clinical diagnosis made during the evaluation itself. Thus, a functional deficits-based rehabilitation program that addresses both the clinical entity and the functional status of the shoulder will be prescribed.

The patient will complete the initial clinical and functional evaluation in the medical room and the laboratory. Afterwards, all the gathered information will be assembled to generate the patient-specific functional (i.e., related to the biomechanical laboratory examination) and clinical (i.e., related to the physician and laboratory examinations) status-based rehabilitation program. This program will be generated in conjunction with the physical therapy staff (Table 1). For each of the functional results (i.e., those related to force deficits or movement attenuation complaints) or clinical status deficits (i.e., those related to positive results on the orthopedic tests) identified during the examination, the physical therapist will identify the precise exercise and goal-based progression using a previously standardized goal-based rehabilitation algorithm adapted from previously published investigations targeting this issue [17, 18, 21] (Additional files 1, 2, 3 and 4).

### Outcome measures

#### Primary outcome

##### Pain

Pain levels during the complete clinical and functional examinations will be recorded using the previously validated visual analogue scale (VAS) [22] at the time of the functional examination in the laboratory setting. This qualitative test uses a 10-cm graded horizontal line ranging from 0 to 10 points. A 0-point score represents no pain at all, and a 10-point score is assigned to the worst pain ever felt. Immediately after each of the tests performed during the laboratory functional screening examination, the patient will be asked to use the scale to indicate the level of pain he or she experienced.

##### Range of motion measurement

The shoulder range of motion (ROM) will be measured using 3 iso-inertial unit STT-IBS (STT Systems, San Sebastian, Spain)-based technology.

The STT-IBS^©^ is a 9-degrees-of-freedom inertial measurement unit that integrates an accelerometer, a gyroscope and a magnetometer in each of its axes. The system measures the relative orientation, acceleration and position (along the X, Y, Z axes) of the STT-IBS^©^ sensors and sends this information to a computer with a Bluetooth-enabled host. The raw signals are processed online using iSens software (STT-Systems^©^, Spain), which provides the angular velocity, the acceleration and the angular position of each STT-IBS. Furthermore, after the preferred model is selected (i.e., flexion/extension, FLX/EXT shoulder model) and the sensor units are placed accordingly, the software provides the angular measurement of the selected movement in each plane.

After determining a reference position or zero position, the software measures this angular position as the projection of the vector position of each sensor in the corresponding plane determined by the reference sensor (i.e., the FLEX/EXT movement is measured as the projection of the position vector of the arm’s sensor in the sagittal plane determined by the sensor on the back).

**Fig. 2:**
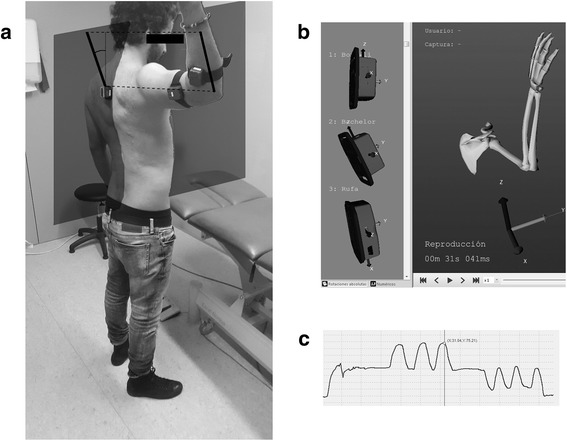
Range of Movement (ROM) measurement during the internal and external rotation evaluation using the inertial sensors. **a** Placement of the sensors (the reference sensor between the scapulas, and the others in the arm and forearm) and representation of the calculation of the angle as the projection in the sagittal plane. **b** Analysis software. **c** Example of ROM-Time curve

The 3 STT-IBS units will be placed on the arm and forearm of the upper limb with straps (moving sensors) and at the patient’s inter-scapular level (reference sensor) with double-sided tape according to the specifications of the models provided by the manufacturer (STT-Systems, Spain; Fig. 2). The flexion/extension (FLX/EXT), abduction/adduction (ABD-ADD) and internal/external rotation (IR/ER) movements of the affected shoulder will be measured using the corresponding models.

The first movement tested will be FLX/EXT. The patient will be asked to perform 3 maximal FLX movements starting from a neutral position (with the arm relaxed and aligned with the axial direction of the body). Three consecutive FLX and EXT movements starting from a neutral position will be performed. Next, the ABD movement will be measured. Similar to the previous movement, the patient will be asked to perform 3 consecutive ABD maneuvers, returning to a neutral position between repetitions.

**Fig. 3:**
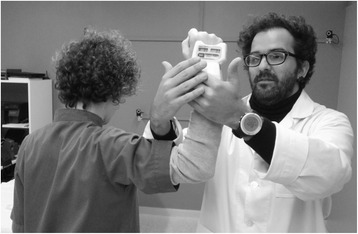
Example of a orthopedic test measurement using a dynamometer (Microfet 3, Hoggan Health)

Finally, the IR/ER task will be analyzed. The patients will be positioned with the arm parallel to the ground (90°ABD), with the forearm perpendicular to the arm and the ground and parallel to the trunk (Fig. 3). When ready, the patient will be asked to perform 3 consecutive IR and ER movements, returning to the initial neutral position after each repetition. If the patient does not return to the initial position between maneuvers, the test will be considered invalid.

Data analysis will be performed offline. The raw angular data of each pair of movements (i.e., flexion/extension movement) will be exported to an Excel file (Microsoft Office 2010, USA), and the maximum and minimum values will be obtained. The maximum values correspond to the FLX, ABD and IR movements. Conversely, the minimum values correspond to the EXT, ADD and ER. The mean of 3 repetitions for each movement will be obtained for further statistical analysis.

The shoulder range of motion (ROM) will be measured using 3 iso-inertial unit STT-IBS (STT Systems, San Sebastian, Spain)-based technology.

The STT-IBS^©^ is a 9-degrees-of-freedom inertial measurement unit that integrates an accelerometer, a gyroscope and a magnetometer in each of its axes. The system measures the relative orientation, acceleration and position (along the X, Y, Z axes) of the STT-IBS^©^ sensors and sends this information to a computer with a Bluetooth-enabled host. The raw signals are processed online using iSens software (STT-Systems^©^, Spain), which provides the angular velocity, the acceleration and the angular position of each STT-IBS. Furthermore, after the preferred model is selected (i.e., flexion/extension, FLX/EXT shoulder model) and the sensor units are placed accordingly, the software provides the angular measurement of the selected movement in each plane.

After determining a reference position or zero position, the software measures this angular position as the projection of the vector position of each sensor in the corresponding plane determined by the reference sensor (i.e., the FLEX/EXT movement is measured as the projection of the position vector of the arm’s sensor in the sagittal plane determined by the sensor on the back).

The 3 STT-IBS units will be placed on the arm and forearm of the upper limb with straps (moving sensors) and at the patient’s inter-scapular level (reference sensor) with double-sided tape according to the specifications of the models provided by the manufacturer (STT-Systems, Spain; Fig. 2). The flexion/extension (FLX/EXT), abduction/adduction (ABD-ADD) and internal/external rotation (IR/ER) movements of the affected shoulder will be measured using the corresponding models.

The first movement tested will be FLX/EXT. The patient will be asked to perform 3 maximal FLX movements starting from a neutral position (with the arm relaxed and aligned with the axial direction of the body). Three consecutive FLX and EXT movements starting from a neutral position will be performed. Next, the ABD movement will be measured. Similar to the previous movement, the patient will be asked to perform 3 consecutive ABD maneuvers, returning to a neutral position between repetitions.

Finally, the IR/ER task will be analyzed. The patients will be positioned with the arm parallel to the ground (90°ABD), with the forearm perpendicular to the arm and the ground and parallel to the trunk (Fig. 2). When ready, the patient will be asked to perform 3 consecutive IR and ER movements, returning to the initial neutral position after each repetition. If the patient does not return to the initial position between maneuvers, the test will be considered invalid.

Data analysis will be performed offline. The raw angular data of each pair of movements (i.e., flexion/extension movement) will be exported to an Excel file (Microsoft Office 2010, USA), and the maximum and minimum values will be obtained. The maximum values correspond to the FLX, ABD and IR movements. Conversely, the minimum values correspond to the EXT, ADD and ER. The mean of 3 repetitions for each movement will be obtained for further statistical analysis.

##### Isometric peak force evaluation

Throughout the screening examination, each of the orthopedic tests will be performed using a hand-held dynamometer (MicroFeet 1 Hoogan Industries, USA) to register the peak force (N) exerted during each task in addition to the standard clinical interpretation of the pain elicited during the maneuver. Three repetitions will be performed for each limb at each testing position. The first repetition will serve as familiarization, whereas the subsequent two repetitions will be recorded for further analysis.

#### Secondary outcome measures

##### Measures of self-reported shoulder function

Self-reported shoulder function will be registered using the Simple Shoulder Test Questionnaire (SST). The questionnaire consists of 12 items answered with dichotomous responses (yes/no). Two questions are related to pain, 7 are related to function and strength, and 3 are related to range of motion perceptions. The minimum clinically important difference between pre- and post-rehabilitation evaluations was set at 2 to 2.33 points [23]. The questionnaires will be completed each time the patient undergoes a functional evaluation at the laboratory.

##### Cost effectiveness analysis

When the study concludes, the number of rehabilitation and medical visits administered and the number of working days lost among the participants will be analyzed. Thus, the economic burden of the rehabilitative process (including both medical costs and the costs of missed work) will be determined for comparison with historical cohorts from the same medical institution (60 patients recruited the year before the OCBRA model is implemented). The number of overall rehabilitation and medical visits made will by multiplied by their typical economic costs at the hosting medical institution. The number of sessions administered and the number of work days missed will be provided by the mutual insurance society for work-related injury management. The economic burden of both medical costs and missed work days will be provided by the same institution following the recommendations of the National Social Health Agency of the Spanish Heath Service Ministry. The cost effectiveness ratio will be calculated as the number of visits + missed work days divided by the sum of the economic burdens.

### Statistics and sample size

Considering the multi-factorial nature of the present study’s design, statistical power should be adjusted (and sufficient) for each of the primary outcomes analyzed. A minimum of 80% statistical power will be guaranteed for all of the primary outcome variables [2]. Thus, as Ginn et al. [2] established, a sample size of 70 patients would be sufficient for a statistical power of 0.80 for a shoulder abduction change of 25° over time. Such an effect of the physical rehabilitation program is believed to be realistic. For force application-related changes over time as a result of the rehabilitation program, Bang et al. [24] also used a multi-factorial evaluation design to assess shoulder rehabilitation program efficacy and allocated 55 patients into two different rehabilitation groups. Thus, 46 subjects were enrolled in each group. Based on this previous study [2], our study protocol will recruit 120 patients (60 undergoing the proposed model of injury management and 60 patients who were previously treated using the previous routine care protocol and identified as clinical cohorts), thus ensuring a sufficient sample size to account for potential drop-outs in the intervention group based on formulae previously described by Whitley et al. [25].

$$ n=\frac{2}{d^2}{c}_{p, power} $$

Where *n* is the number of patients required in each group, *d* is the standardized difference and *c*_*p,power*_ is a constant defined by the values chosen for the *P* (0.05) value and power (0.8) for this study, resulting on a constant value of 7.9.

In relation to statistical analysis of the collected variables, descriptive statistics and normality (Shapiro Wilk test) and variance homogeneity (Levene test) tests will be performed for intra-group analysis (patients recruited in the proposed injury management model). Perceived changes will be compared using cross tabulations with *X* ^2^ analysis. Following logarithmic transformation if required, mean changes in pain intensity, functional limitation (Simple Shoulder Test questionnaire), ROM and force application capability will be compared using a repeated measures paired *T*-Test. All analysis will be performed with SPSS^©^ (statistical software package for social sciences v 22.0 Chicago, IL, USA). The level of statistical significance will be set at *p* < 0.05. Simultaneously, the magnitude of the treatment effect will be tested by the determination of the effect size (Cohen’s *d*). This analysis will be performed as the standardized difference between two means (pre and post rehabilitation program). Cohen’s standard attributes and effect size for the intervention based on the magnitude of the observed size will be described as a large effect size for 0.8 or greater, medium for 0.5 to 0.8 and small for 0.2 or less [26].

## Discussion

The present study protocol aims to complement previously published shoulder injury management algorithms. The proposed objective criteria-based rehabilitation algorithm (OCBRA) focuses on the clinical entity of the examined shoulder and its functional status with respect to the joint range of motion, the force exertion capability and the pain elicited during the evaluation. Thus, the prescribed rehabilitation program is designed to treat the clinically observed affectation and the actual and quantitative deficits identified through a standardized functional evaluation.

Hegedus et al. [9] recently published a notable systematic review with a meta-analysis addressing the most valuable physical examination test for shoulder injury. The authors found important methodological implications regarding commonly used orthopedic tests and determined that actual diagnostic accuracy values and further high-quality research in this field are still required. Other investigations have also supported this need [10].

In relation to exercise protocols for shoulder injuries, a wide source of scientific investigations has addressed EMG activity [12, 27, 28], biomechanical injury [18, 29, 30] and pathology-related [8, 13, 31, 32] concerns in relation to commonly reproduced shoulder injuries. These guides are very helpful during the rehabilitation process for clinicians who prescribe treatments and manage patients with shoulder injuries.

However, in the authors’ opinion, this is the first study aiming to fuse the clinical entity with an objective and standardized functional evaluation and a coordinated (specific functional- and clinical results-based) rehabilitation program to help patients fully recover from multi-etiological shoulder complaints. If this new rehabilitation model proves to be useful, it could be instituted by several health companies to improve shoulder injury management routines, thereby hastening patients’ return to previous activity levels and reducing the economic burden associated with these pathological conditions.

### Trial status

The trial will start recruitment on January 2017. The trial will remain undecidedly open given that the present intervention program is the standard for shoulder injury care at the friendly society for work-related injuries management wherein the study is being performed. Once 100 patients are enrolled, a case- series retrospective study will be performed to compare the cost efficiency in terms of time duration, number of physical therapy and physician visits perceived and economical burden of the present shoulder injury management program vs. historical cohorts of patients receiving care at our institution.

## Additional files

Additional file 1: (Blue). Example of scapulothoracic instability exercises. The complexity of the exercises increases from a to b. The patient must control scapular retraction (a) and co-contraction from scapular retractor and protractors (b). (TIF 482 kb)
Additional file 2: (Pink). Examples of internal rotation deficit exercises. The complexity of the exercises increases from (a) to (c). The patient imposes different stretching intensities to the shoulder posterior capsule depending on his/her tolerance. (TIF 563 kb)
Additional file 3: (Red). Examples of exercises to improve strength deficits. The complexity of the exercises increases from (a) to (c). Progression is made in order to isolate different rotator cuff muscles isolated (a, b) or in combination to the scapular retractors (c). (TIF 689 kb)
Additional file 4: (Green). Examples of glenohumeral instability exercises. The complexity of the exercises increases from a to c. Progression is made from discharged closed (a,b) to closed full weight bearing unstable surfaces. (TIF 800 kb)

## Abbreviation

ABD-ADD: Abduction-adduction
EMG: Electromyography
FLEX-EXT: Flexion-extension
GH: Gleno-humeral
IR-ER: Internal-external rotation
OCBRA: Objective criteria based rehabilitation algorithm
ROM: Range of motion
SIS: Subacromial impingement syndrome
SLAP: Superior labroum anterior-posterior
VAS: Visual analogue scale
